# Screening of a core SNP set for the seedling identification of *Saccharina japonica* cultivars in northern China

**DOI:** 10.1371/journal.pone.0324295

**Published:** 2025-06-03

**Authors:** Xiangyu Wang, Aihuan Song, Wenxi Zhao, Xiaohui Liu, Fang Lü

**Affiliations:** Marine Science Research Institute of Shandong Province, Qingdao, Shandong, China; National Cheng Kung University, TAIWAN

## Abstract

Kelp is an economically important seaweed species in China. The verification of *Saccharina japonica* cultivars is crucial for industrial development. Four *Saccharina japonica* cultivars with distinctive characteristics that are widely used in China were selected. To distinguish these cultivars, a highly informative core SNP marker set was identified and validated from 441,094 SNPs. The marker set consisted of 247 core SNPs that were uniformly distributed and were abundant polymorphisms. The similarity between samples was calculated using both core and total SNPs, and a significant linear correlation (P < 0.01) was observed, with a correlation coefficient of *r* = 0.99. These results provide a valuable SNP set for distinguishing the germplasms of four *Saccharina japonica* cultivars and serve as a reference for variety differentiation.

## Introduction

*Saccharina japonica* (Areschoug) C.E. Lane, C. Mayes, Druehl and G. W. Saunders (Laminariales, Phaeophyceae) is a seaweed species that is naturally distributed in the northwest Pacific Ocean [1]. It exhibits alternation of generations, including a tiny gametophyte generation and a large sporophyte generation, with the sporophyte generation accounting for most of its life cycle [2]. Under favorable conditions, gametophytes can grow vegetatively and form longer filaments that can be preserved for a long time [3]. On the other hand, sporophytes can be harvested and used for food and as a raw material for chemical production [4]. However, the demand for wild kelp resources is too high to be met naturally, so countries such as Japan, Korea, and China have started cultivating kelp [5]. In the 1920s, *Saccharina japonica* was introduced in China for large-scale cultivation [6]. Cultivar selection has also been carried out [7], resulting in high-quality *Saccharina japonica* cultivars that contribute to China’s position as the world’s top producer of this seaweed, meeting market demand. There are many varieties or strains overall, but many strains have lost their unique traits in actual production because of the lack of effective purification techniques [8]. China has 18400 km of continental coastline, with highly diverse marine environments [9], and kelp cultivation is concentrated from south to north in the subtropical Fujian Province, warm temperate Shandong Province and temperate Liaoning Province The kelp in these three regions has developed more specialized stability-related traits due to long-term geographic adaptations; for example, Fujian kelp is wide and short, with an inconspicuous mid-rib, and does not decay at relatively high temperatures. Shandong and Liaoning kelps are characterized by being longer, and their midribs are more prominent. Kelp cultivars from these areas have remarkable and stable characteristics at the adult stage, are adapted to the local cultivation environment and are the main cultivars in each of the main cultivation areas.

In China, the seedlings needed for cultivation come mainly from specialized kelp nurseries. The nurseries are concentrated in Fujian Province in southern China and Shandong Province in northern China. Shandong Province produces 8.9 billion plants, accounting for 26% of China’s total production [10], and the seedlings used for kelp farming in Shandong also include the main cultivars from all three regions. These seedlings have some similar traits, especially at the seedling stage, when trait differentiation is not obvious. First, kelp in China is derived from *Saccharina japonica* [11], and there is a kinship among the cultivars [12]. Moreover, seedling nurseries adopt industrial methods for rearing kelp in natural light and low-temperature water systems [13]. To meet diverse market needs, nurseries often breed more than two cultivars under the same rearing conditions. The seedlings grow in the same recirculating water system, and the spores or sperm may be mixed [14], inevitably, cultivar mixing occurs. The identification of cultivars is difficult because of these problems. Therefore, it is necessary to develop a technology for identifying *Saccharina japonica* cultivars. However, the identification of seedlings of *Saccharina* cultivars are scarce.

DNA molecular markers are highly sensitive and highly polymorphic, making them effective at accurately detecting varietal differences [15], which have been used to identify a variety of higher plants [16–18]. Mitogenomic DNA barcoding technology has been used to identify low-level seaweed species [19–21]. Random amplified polymorphic DNA (RAPD) was used by He [22] and Wang [23] to construct fingerprints of *Saccharina* species, with gametophyte filaments used as experimental materials. Single-nucleotide polymorphism (SNP) markers are also molecular markers of DNA. Compared with other markers, SNPs have several advantages, such as low mutation rates, fewer variations, higher genetic stability, and greater gene polymorphism [24]. Because of these characteristics, SNPs are particularly useful for identifying Chinese kelp cultivars with similar genetic backgrounds. Genotyping by sequencing (GBS) is a rapid, straightforward, and cost-effective method [25]. This method uses next-generation sequencing (NGS) to identify SNPs. GBS has been used extensively to identify various plant varieties [26–29]. The main aim of this study was to identify a set of SNPs using the GBS approach capable of distinguishing seedlings of the main kelp cultivars in northern China. This will lay the technical foundation for the design of primers for the core markers to be used in production practice, and this low-cost and high-precision technology can be used for further kelp germplasm identification in China.

## Materials and methods

### *Saccharina japonica* cultivars

For this study, for NGS and core SNPs detection, we selected the seedlings of four main *Saccharina japonica* cultivars from Shandong Province with distinct and stable traits (listed in Table 1). Each cultivar comprised ten healthy seedlings. HG is a nationally certified variety widely cultivated in the main production areas of China [30], and bred in Lianjiang city, Fujian Province. ZK1 and ZK2 are representative cultivars from Rongcheng city, Shandong Province, whereas ZK1 and B013 [31] are representative cultivars from Changdao city, Shandong Province. All three cultivars, ZK1, ZK2 and B013 were bred in Rongcheng city.

**Table 1:** Information on *Saccharina japonica* cultivars.

Cultivar	Sampling site	Coordinate	Main characters
**HG**	Guanwu, Lianjiang	119.80°/26.28°	flat frond middle band, narrow frond margin, high temperature resistance [32]
**B013**	Lijiang, Rongcheng	122.51°/37.16°	dark green coloration of the dried product, high iodine content [33]
**ZK1**	Lidao, Rongcheng	122.49°/37.17°	wide frond, narrow frond margin, dark brown [34]
**ZK2**	Lidao, Rongcheng	122.49°/37.17°	wide frond, narrow frond margin, dark brown, no yellow or white margin [35]

### DNA extraction

Young brown seedlings, approximately 4–6 cm in length, were collected and frozen on dry ice. Genomic DNA was extracted using a Plant Genomic DNA Kit (TIANGEN BIOTECH CO., LTD). The purity and concentration of the extracted DNA were determined using a NanoDrop 2000 spectrophotometer. Forty high-quality DNA samples were obtained, which were then diluted to a concentration of 50 ng/μL.

### High-throughput sequencing and data processing

High-throughput sequencing is an individual sequencing method. The genomic DNA was genotyped using the GBS method [36]. In a 30 µl reaction, 200 ng of DNA was digested with the *Pst* I*-*HF-*Msp* restriction enzyme at 37 °C for 2 h. The enzymes were then inactivated by incubation for 20 min at 75 °C. Ligation was performed at 22 °C for 2 h in a 40 μL reaction containing 20 μL of restriction digest, 1 μL of barcoded *Pst* I-HF adaptor (stock:0.1 μM), 1.5 μL of the common *Msp* I adaptor, 4 μL of 10X T4 DNA ligase reaction buffer, and 200 U of T4 DNA ligase.

At room temperature, DNA fragments smaller than 300 bp were removed by incubating the samples with 0.7 volumes of Sera-Mag SpeedBeads (GE Healthcare Life Sciences) for 5 min. The beads were then separated using a magnetic stand and washed three times with 200 μL of 70% ethanol. DNA was eluted from air-dried beads with 40 μL 10 of mM Tris-HCl (pH 8.0). Three microliters of eluent were added to a mixture of 16 μL of H_2_O, 5 μL of 5X Taq master mix, 0.5 μL of forward primer specific to the barcoded adaptor (stock: 10 μM) and 0.5 μL of reverse primer homologous to the common adaptor (stock:10 μM).

PCR amplification was performed separately for each sample using high-fidelity DNA polymerase. The reaction conditions were as follows: initial denaturation at 95 °C for 30 s, followed by 16 cycles of denaturation at 95 °C for 30 s, primer annealing at 62 °C for 20 s, and fragment elongation at 68 °C for 15 s, and a final elongation step at 68 °C for 5 min. Eight microliters of the PCR product was analyzed on a 1.5% agarose gel. The DNA concentration of each GBS library was measured using a Qubit 2.0 and a Qubit™ dsDNA HS Assay Kit. GBS libraries with concentrations > 5.0 ng/μL were sequenced. The concentration of each GBS library pool was 30 ng/ml. The primers, dNTP, and small DNA fragments were removed from the DNA pool using 0.7 volume Sera-Mag SpeedBeads. GBS libraries (100 ng) were sequenced on an Illumina platform (Illumina Nova 6000, PE150) with 150 bp paired end reads. Read quality was assessed via the QC and GC contents. GBS was performed at Qingdao OE Biotech Co., Ltd. (Qingdao, China).

### Identification of SNPs

FastQC [37] (v0.11.7) was used to check the read quality. The reads were split by barcode using the “process_radtags” module in the “Stacks” [38] program (v2.1), with the options -r --renz_1 --adapter_mm 1. Forward reads passing the filter carried both the barcode and *Pst* I restriction sites. “FASTX_trimmer” in the “FASTX Toolkit” [39] (v0.0.14) package was used to remove restriction sites as well as all bases at the 3′ end of reads with FastQC quality scores below 20. Clean reads were compared to genomic references (NCBI: GCA_000978595.1) using Bowtie2 [40] (v2.3.4.1) and default parameters (--maxins 1000 --no-discordant --no-mixed). GATK [41] (v3.8-1) was used to identify all variants, including SNPs and Indels. Variant screening was performed via VCFtools [42] (v0.1.13) with the following criteria: (1) sequencing depth of less than four loci; (2) SNPs with minor allele frequency (MAF) < 0.01 were excluded; and (3) SNPs with deletions in > 20% of the samples were excluded. SnpEff [43] (version 4.1g) was used to annotate the screened SNPs.

### Core SNP identification and fingerprint construction

The goal of developing core SNPs is to distinguish as many samples as possible with as few markers as possible, and the subsequent design of primers for the core SNPs for use in production practice can reduce costs. The screening criteria for core SNPs include high quality, high strain differentiation, and uniform distribution of genomes [44–47].

The obtained SNP loci were screened according to the following steps. The filter standard was modified from the given VCF file to ensure typing accuracy, so only SNP loci with a sequencing depth > 4 and biallelic sites were retained as sample total SNPs; The group normalization standard was modified, and we compared sample genotypes within each variety, reducing the gene identity rate from 100% to 70% to account for possible sample heterogeneity within the same strain. If the gene identity rate was ≥ 70.0%, it was used as the variant genotype at the SNP locus; otherwise, missing data represented the variety genotype. SNP loci without polymorphisms and p value of the

Hardy‒Weinberg equilibrium (HWE) test < 1e^-5^ were eliminated; SNP loci with SNP missingness > 30. 0% and MAF < 0.01 were excluded to obtain high quality typing results for practical applications. SNP loci with polymorphic information content (PIC) < 0.1 were eliminated to ensure effective discriminatory power, and a chain filter with the parameter --indep-pairwise 50 10 0.99 was used to avoid the redundancy of SNPs in PLINK software (1.90p) [48]. The density distribution of total SNPs and core SNPs across the chromosome was investigated using a sliding window approach with both window and step sizes set at 500,000 bp. This analysis was performed based on a chromosome-level genome assembly (GCA_046627945.1). An in-house script was used to plot a similarity correlation diagram between pairs of samples using the R language. A heatmap was drawn based on the core SNP dataset.

### Population analysis

VCF Tools software was used to analyze allele frequency. The observed heterozygosity (*H*o), MAF and nucleotide diversity (Pi) were calculated using PLINK. The PIC was calculated using PIC_CALC 0.6. For genetic analysis, each marker genotype was assembled from head to tail, and missing sites were replaced with “-”.

The neighbor-joining tree [49] was constructed using Treebest [50] (version 1.9.2) with the p-distance model [51] and bootstrapping (1,000). The R package ggtree (v1.16.6) was used to visualize the phylogenetic tree. SNP genotyping information was analyzed using ADMIXTURE [52] (version 1.3.0) to determine the optimal population size (K) and population structure. Principal component analysis (PCA) of the SNPs was performed using GCTA [53] (version 2.0).

## Results

### GBS analysis and SNP marker development and genotyping

After Illumina sequencing of 40 *Saccharina japonica* cultivars, a total of 25.71 Gb of raw data was produced comprising 0.175 billion reads. After filtering, the data were cleaned, producing 24.45 Gb of clean data and 0.168 billion clean reads, with a 95.10% raw read conversion rate and an average of 4.19 million reads per sample.

**Fig 1:**
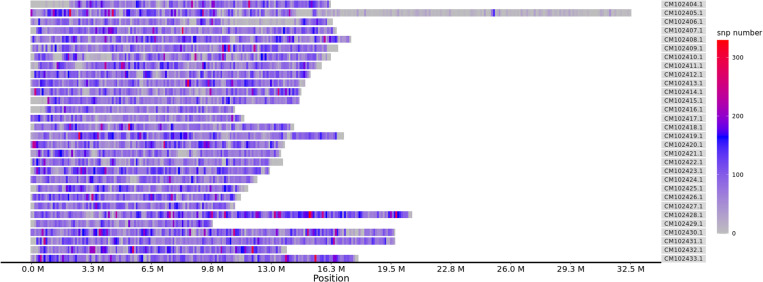
Total SNP density distribution maps. This figure represents the distribution of total SNPs across the genome. Abscissa: physical location of the chromosome, ordinate: GenBank accession number of chromosome, color: number of SNPs in this window.

The sequencing data quality was predominantly high, with Q20 and Q30 values ranging from 95.92% to 97.19% and 89.61% to 92.33%, respectively, and mean values of 96.80% and 91.43%, respectively. The guanine-cytosine (GC) content ranged from 49.69% to 50.54%, with a mean of 50.11%. These results indicated the reliability and quality of the DNA sequencing data for subsequent analyses (S1 Table). The mapping rate (the total number of reads compared with the reference genome) ranged from 91.27% to 98.23%, and the average coverage depth for the reference genome was 11.61 × , with a mean coverage depth ranging from 10.16% to 9.95% (S2 Table). After quality control and removal of SNPs with outliers, 441,094 high-quality biallelic SNPs were identified (S3 Table), and they were distributed throughout the genome (GCA_046627945.1). (Fig 1).

### Selection and validation of core SNPs

**Fig 2:**
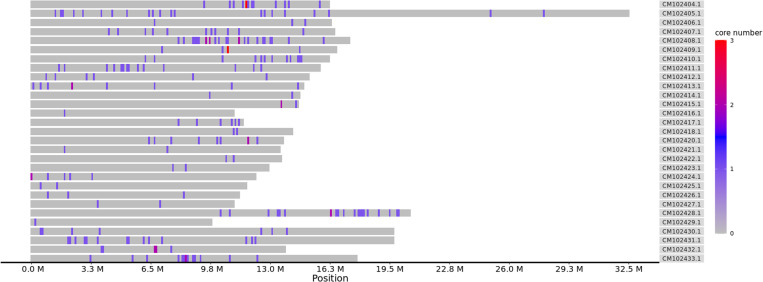
Core SNP density distribution maps. This figure represents the distribution of Core SNPs across the genome.

**Fig 3:**
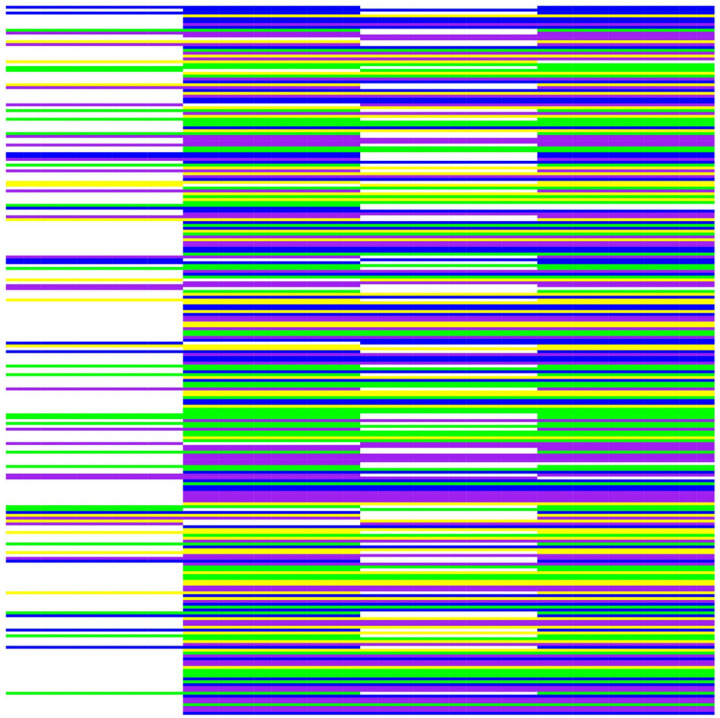
Core SNP heatmap of *Saccharina* germplasms. Each row represents an SNP locus, and each column represents a sample. The colors for CC, AA, TT, and GG are yellow, green, blue, and purple, respectively. Missing data are displayed in gray, and heterozygosity data are displayed in white. Shown from left to right are B013, HG, ZK1, and ZK2.

A total of 247 high-quality SNPs distributed on 31 chromosomes of the genome (GCA_046627945.1) (Fig 2) were identified as core SNPs (S4 Table). These core SNPs exhibited high polymorphism, good reproducibility, easy resolution of two-dimensional phenotypes, and high specificity (Fig 3).

Abscissa: physical location of the chromosome, ordinate: GenBank accession number of chromosomes, color: number of SNPs in this window.

### Population structure

**Fig 4:**
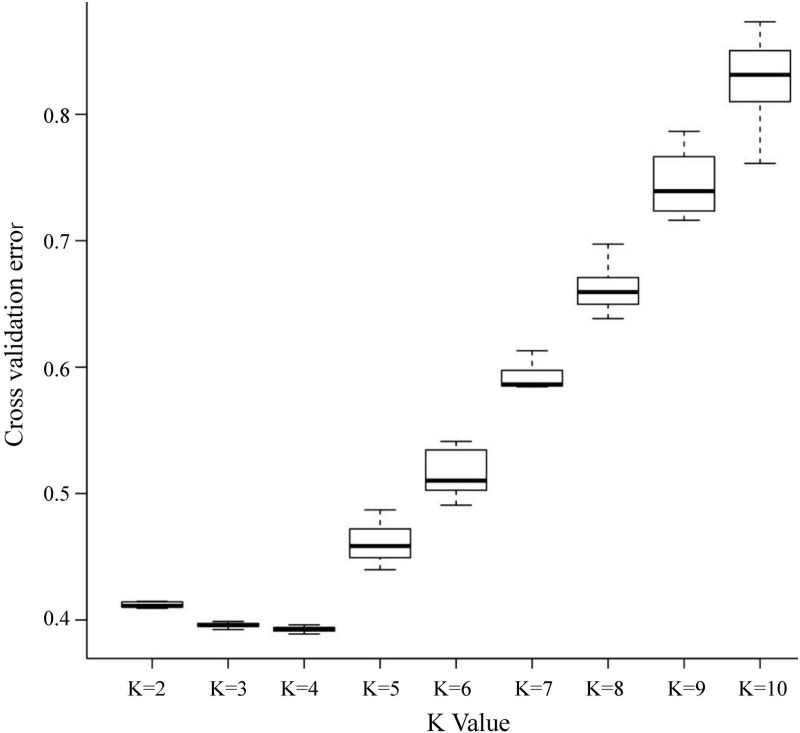
CV error and K based on total SNPs.

**Fig 5:**
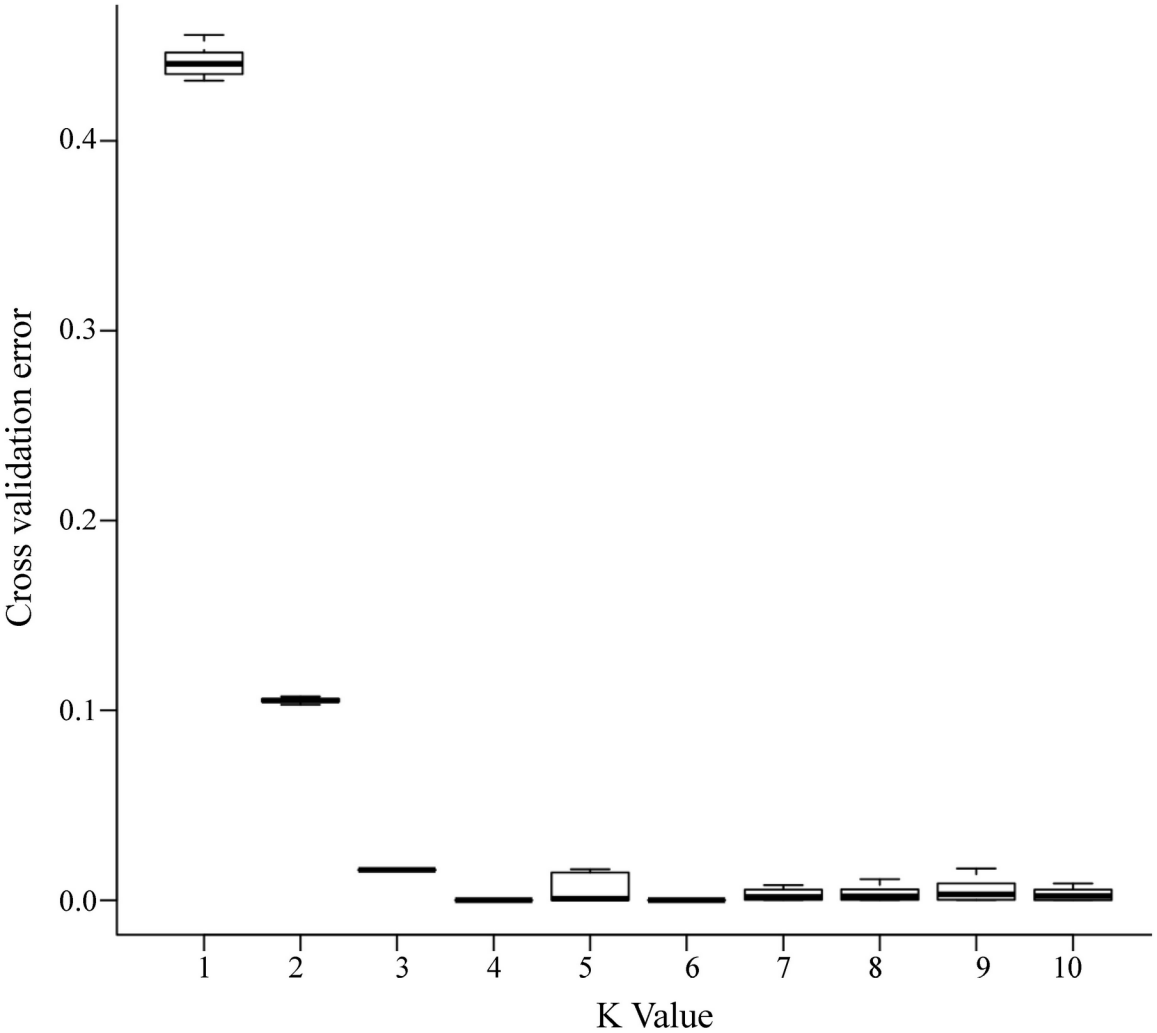
CV error and K based on core SNPs.

The genetic structure of 40 *Saccharina* samples from the four populations was analyzed on the basis of the total SNP and core SNP typing data. The coefficient of variation (CV) error of the total SNPs was assessed for K values ranging from 2 to 10, and the CV error was the lowest when K = 4, as shown in Fig 4. Similarly, the CV error of core SNPs was assessed for K values ranging from 1 to 10, and the CV error was the lowest when K = 4, as shown in Fig 5. The structure analysis results for the core SNPs were consistent with those for the total SNPs.

### PCA and cluster analysis

**Fig 6:**
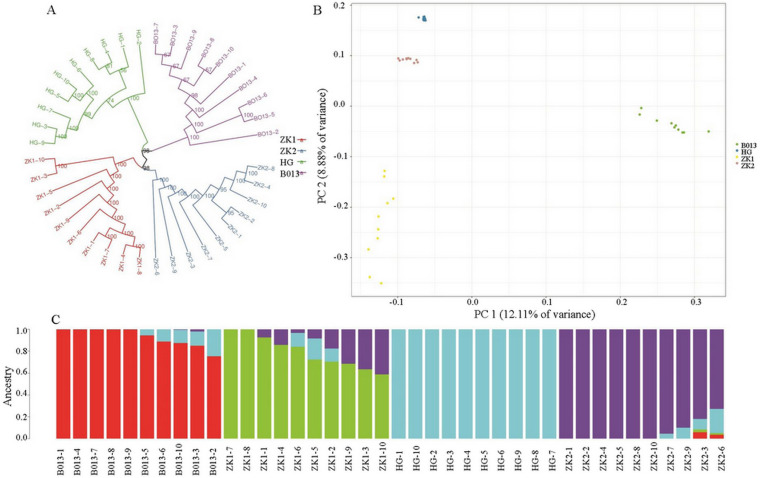
Phylogenetic tree (A), PCA (B), and structure (C) base on the total SNPs.

**Fig 7:**
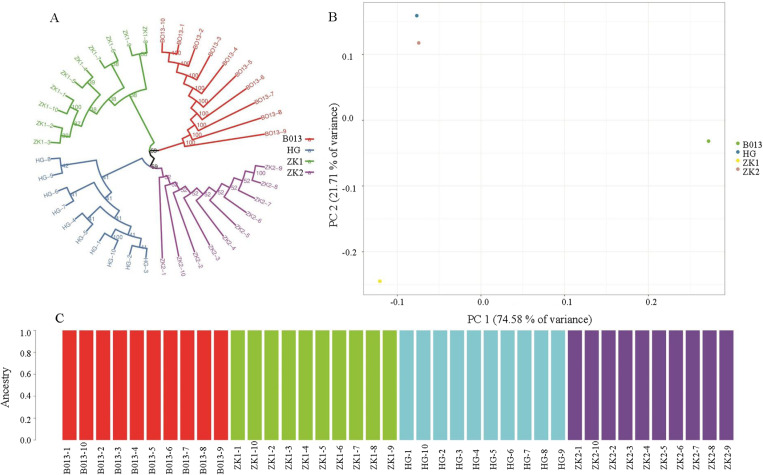
Phylogenetic tree (A), PCA (B), and structure (C) base on the core SNPs.

To validate the population analysis results obtained from the structure, PCA was performed on the total SNPs, core SNPs, and first and second SNPs. The principal components were then calculated. In the total SNP analysis, the first and second principal components contributed 12.11% and 8.88% of the total variation, respectively. In the core SNP analysis, the first and second principal components contributed 74.58% and 21.71% of the total variation, respectively. PCA based on core SNPs yielded higher percentages of PCA1 and PCA2, indicating greater discriminatory power for the samples. The 4 cultivars were divided into four groups on the basis of their genotypic materials, with cultivars of different genotypes clustered together. The phylogenetic tree clearly revealed population differentiation among the different cultivars (Fig 6 and Fig 7), and the results were consistent with those of the PCA and structure analysis.

### Population genetic diversity analysis

**Fig 8:**
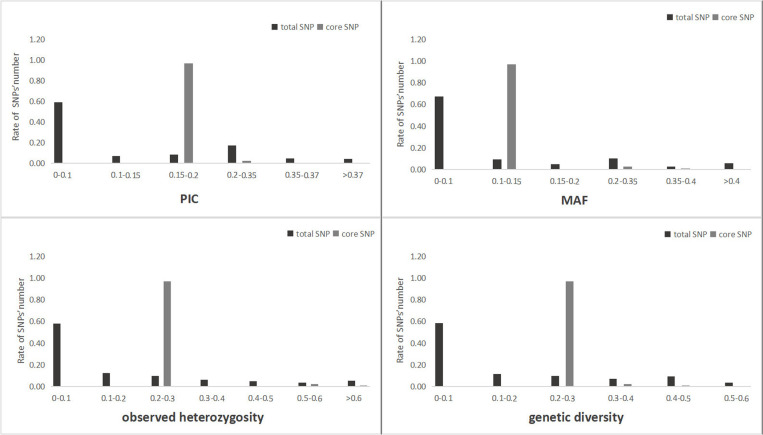
Population genetic analysis of *Saccharina japonica* cultivar accessions on the basis of the total SNPs and core SNPs.

Genetic diversity analysis was performed using the developed SNPs; the core SNPs presented better polymorphism and variety discrimination ability. The mean PIC value of the total SNPs (S5 Table), was 0.122, ranging from 0.024 to 0.375; the mean MAF value was 0.099, ranging from 0.125 to 0.500; the mean *H*o value was 0.16, ranging from 0 to 1; and, the mean genetic diversity value was 0.15, ranging from 0.03 to 0.51. For the core SNPs (S6 Table), the mean PIC value was 0.199, ranging from 0.195 to 0.359; the mean MAF value was 0.130, ranging from 0.125 to 0.375; the mean *H*o value was 0.26, ranging from 0.25 to 0.75; and the mean genetic diversity value was 0.23, ranging from 0.22 to 0.47. These values were higher than those calculated on the basis of the total SNPs (Fig 8).

### Similarity correlation analysis

**Fig 9:**
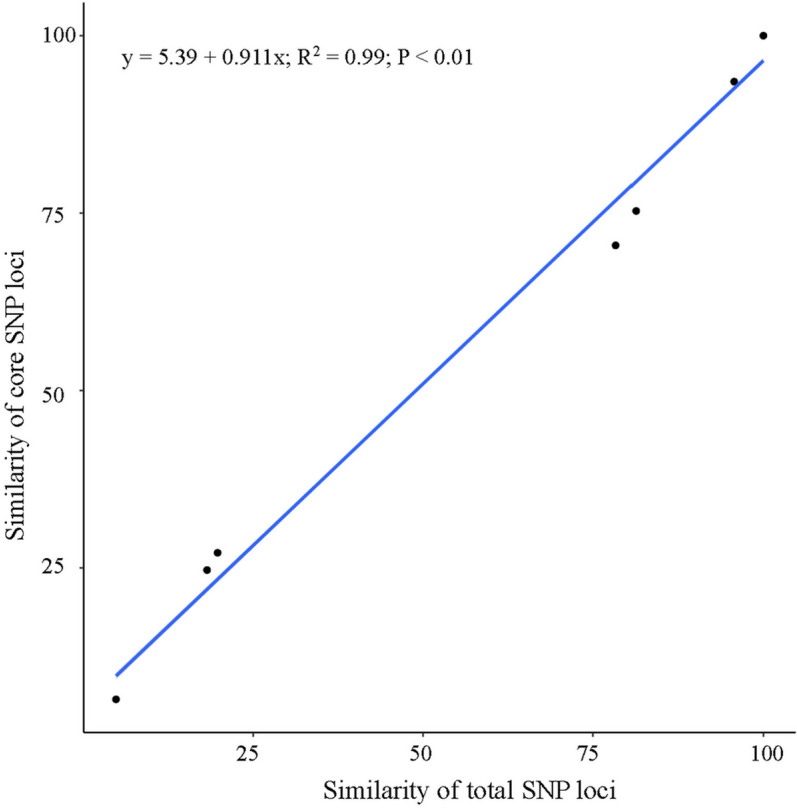
Similarity correlation of total SNP loci and core SNP loci. Abscissa: similarity calculated using all sites; ordinate: similarity calculated using the core site; upper left corner: fitted curve formula, correlation coefficient and p value.

The similarity between two samples was determined based on the total SNP and core SNP datasets of each sample (Fig 9). The formula for calculating similarity was as follows: the number of SNPs with consistent typing divided by the total number of common SNPs. Using the ggplot2 package in R, we plotted the data, added regression lines, fitted linear models, and calculated Pearson coefficients and P values on the basis of the similarity between the two datasets. Similarity correlation analysis revealed a significant linear correlation (P < 0.01) between the similarity of samples calculated using core SNPs and that calculated using total SNPs. The correlation coefficient was *r* = 0.99, indicating that the core SNPs are represented and can be effectively used for identification of the *Saccharina japonica* cultivar.

## Discussion

Currently, China has the greatest number of *Saccharina japonica* cultivars in the world, and for this study, 4 representative strains that are widely used were selected. Among them, B013 is a hybrid cultivar with Rongcheng cultured kelp as the male parent and wild kelp from South Korea as the female parent. Directional selection was carried out after hybridization, resulting in superior color quality and a high dry to fresh weight ratio. HG is a self-crossing cultivar obtained through six consecutive generations of directional breeding from the cultivated population in Lianjiang city, Fujian Province. The blade of this cultivar is flat and wide, and the sporophyte has high-temperature resistance and strong decay resistance with a long growth period. ZK1 is a hybrid cultivar with a flat and wide blade and a narrow edge, while ZK2 is also a hybrid cultivar with a longer and wider blade, no yellow or white edge, and even thickness. As suggested by the above discussion, the sporophyte characteristics have some similarities, making it difficult to distinguish different cultivars, especially at the seedling stage.

The culture environment also significantly influences sporophyte characteristics. However, molecular marker-based techniques are highly effective for germplasm identification because they identify codominance, produce highly reproducible results, and have less environmental impact than techniques based on apparent morphological characteristics. In the past, molecular markers were used to identify *Saccharina* germplasms but were applied to gametophytes of different species [22,23]. Recently, researchers have used SSR molecular markers to analyze the genetic material of *Saccharina* germplasms [54], specifically, *S*.*longissima* and *S*.*angustata* strains with relatively close genetic backgrounds. Additionally, Hwang [55] developed 6 SSR markers to distinguish the Korean *Saccharina* Sugwawon 301 from the Chinese *Saccharina* Huangguan 1. Compared with SSRs, SNPs are more abundant and stable genetic variations in the genome. This study revealed that GBS technology could be used to distinguish 4 *Saccharina japonica* cultivar seedlings accurately via the use of 247 SNP markers, and the number of core markers was lower. This molecular marker technology can be used to identify *Saccharina japonica* cultivars with a close genetic relationship. Among the 4 cultivars investigated, HG is from Lianjiang city, Fujian Province, and is a derivative of the *Saccharina* cultivar found in southern China. The parents used to breed of HK1, HK2, and B013 are also *Saccharina japonica* cultivars but are from northern China. HK1 and HK2 were both bred in the same cultivation area, suggesting that the technology used in this study can be applied to distinguish kelp culture groups. Compared with that in various higher plants, such as bottle gourd [56] and maize [57], the number of SNPs identified in this research is still relatively high. This is due to the high heterozygosity of kelp and the similar genetic backgrounds of *Saccharina* varieties in China.

The results of this study can be further developed in two ways. First, the technology can be optimized to reduce costs at the industrial scale. The technology used should be accurate, simple, and affordable [58]. Techniques for SNP development [59] and detection [60] are varied and can be explored in other applications, e.g., suspension array and multiplex PCR techniques can be used for in-depth studies. Second, sequencing data can be used to analyze functional genes that are closely linked to specific functions or traits [61], although this is also dependent on advances in overall molecular biological research on macroalgae, especially with regard to the increased depth and breadth of functional gene resolution in seaweed.

## Conclusions

In this study, four *Saccharina japonica* cultivars that are widely utilized in northern China were analyzed using GBS technology. A total of 441,094 high-quality SNPs were identified. An optimized combination of 247 core SNPs was filtered; this combination could be used to accurately distinguish the four cultivars. The genetic diversity was analyzed on the basis of core SNPs and was found to be greater than that identified on the basis of the total SNPs. Furthermore, highly significant similarity was observed between the total SNPs and core SNPs. This molecular identification system provides technical support for the protection of *Saccharina japonica* cultivars.

## Supporting information

S1 Table Sequencing data statistics. (XLS)

S2 Table Tags statistics. (XLS)

S3 Table SNP density. (XLS)

S4 Table Core SNPs. (XLS)

S5 Table Total SNPs pop_genetic. (XLS)

S6 Table Core SNPs pop_genetic. (XLS)
